# How Did the Media Report on the Great East Japan Earthquake? Objectivity and Emotionality Seeking in Japanese Media Coverage

**DOI:** 10.1371/journal.pone.0125966

**Published:** 2015-05-18

**Authors:** Yukiko Uchida, Chie Kanagawa, Ako Takenishi, Akira Harada, Kiyotake Okawa, Hiromi Yabuno

**Affiliations:** 1 Kokoro Research Center, Kyoto University, Kyoto City, Kyoto, Japan; 2 Faculty of Management, Otemon Gakukun University, Ibaraki City, Osaka, Japan; 3 Hyogo University of Teacher Education, Kato City, Hyogo, Japan; 4 Faculty of Liberal Arts, Teikyo University, Hachioji City, Tokyo, Japan; University of Toledo, UNITED STATES

## Abstract

The Great East Japan Earthquake was a tragic event requiring critical media involvement. Since the media played an important role in conveying factual information, journalists expressed feeling that it was difficult to guarantee the objectivity of their coverage. As media coverage constructs a socio-culturally shared reality among its audience, an examination of the objectivity and emotionality of the contents of the news coverage is needed. In Study 1, we conducted an exploratory content analysis of TV and newspaper coverage from the six month period following the March 11, 2011 disaster, finding that the news media generally reported neutral and objective factual information about the event, with emotionality shown only in the commentary. In order to examine how media coverage was constructed and evaluated by journalists, in Study 2 we conducted an online survey of 115 journalists working for mass media organizations. We found that that the journalists’ orientations tended to be more objective than emotional, which is consistent with the findings of Study 1. However, their evaluations of the objectivity of the published articles were low, especially for the coverage of the nuclear power plant accident, which was an accident of an unprecedented nature. The negative emotions that journalists experienced during their investigations negatively affected subsequent evaluations of the objectivity of their reporting.

## Introduction

*“Reporting on the tsunami-afflicted area was a difficult experience*. *As a journalist*, *I had to report a very sad story*, *but I also think I had to be more objective*.*”*

*“I tried to be fair in my reporting on the Fukushima nuclear power plant accident*, *but it was difficult to fully understand what happened and what it meant*.*”*

### Socio-cultural constructions of “objectivity”

These are personal quotes from journalists who covered the Great East Japan Earthquake in 2011. Most of the journalists expressed feeling that covering this event was much more difficult than their previous experiences as journalists, particularly with regards to “how to guarantee the objectivity of coverage” and “how to manage their own strong negative emotions when covering the disaster.” Once their stories were published, even if the writers had ambiguous feelings about the objectivity of the piece, readers might understand their reporting as a description of “objective facts”. On the other hand, readers might also expect media reports to contain not only objective information, but also emotional information of the type that can strongly affect understandings of the event.

The perception that the world and its events are a “shared reality” is held not only by individuals, but is also institutionalized in everyday practices and public artifacts such as media coverage (see [1–2]). Media accounts are powerful socio-cultural artifacts that both reflect and foster common socio-cultural understandings [1]. This means that the media does not only present objective facts, but also communicates cultural and personal attitudes, values and emotions [1]. Just as Morling and Lamouex [2] approached media coverage as a “cultural product”—a manifestation of psychological tendencies—we need to consider the psychological aspects of the coverage.

News coverage is a dynamically constructed phenomenon that reflects the audience’s points of view (what they would like to know) and the journalists’ points of view (what they would like to report), and both of them are comprised of socio-cultural factors (what is important in a given socio-cultural context or situation) [1]. Most research on the media has been conducted using a macro-level approach that emphasizes socio-cultural factors, but from a current sociological and social psychological perspective, examining individual journalists’ emotions or perceptions about the media coverage could help reveal how news is constructed (i.e., [1, 3]).

### Disaster and the media: Seeking to be objective

In particular, the media focuses on large-scale events and the responses of the audience to both positive events (e.g., the Olympic Games [1]) and negative events (e.g., natural disasters). For such major events, “information needs” are greatly amplified [4–5]. Therefore, an analysis of mass communication about natural disasters is important (e.g., [6]).

The Great East Japan Earthquake was a critical event that attracted international media coverage, partially due to the shocking footage of the tsunami and the disastrous accident at the Fukushima Daiichi nuclear power plant. The media coverage of the earthquake had a psychological impact on people, including those in areas not directly affected by the disaster [7]. Owing to the enormity of the disaster, it was too difficult for individuals to obtain the “accurate and reliable” information that was necessary to regulate their behaviors (e.g., escaping or helping) and emotions (e.g., anxiety) in an atmosphere of increasing anxiety and decreasing trust in government institutions (see [8]). Especially during the first year, people in Japan were struggling to obtain reliable information about the effects of radiation that would help them make decisions about travel and food choices, and also about tsunami victims in the Tohoku area that would help them understand the extent of damage, what kind of help was needed and the victims’ expected levels of resilience (see [9]).

Usually individuals seek information from both the mass media and social networking services (SNSs). During the disaster period, mass media outlets, such as TV and newspapers, played a primary role in providing emergency alert information and risk communication (e.g., [10]), especially for those who could not or do not access SNS services, such as the elderly. Thus, the mass media had a considerable responsibility during the disaster because it plays a key role in risk management [11].

An analysis of media coverage of national emergencies provides an opportunity to understand the process of socio-cultural meaning-making during risk situations (see [12]). Three years after the March 11^th^ triple disaster, the Japanese public still holds mixed opinions about the evacuation of people from the area surrounding the Fukushima Daiichi nuclear plant, as well as the use of nuclear power in Japan as a whole. Such ambivalence is partly due to the “mixed” information that was delivered by the media during this unprecedented situation.

### Objectivity vs. Emotionality in Japanese Culture

We should look not only at the role of the media in risk situations, but also at the socio-cultural orientations found in the media within Japanese contexts. Previous research has suggested that there are sizable cross-cultural differences in how the media portrays events and at what point in the event timeline they begin coverage [13].

One of the features of the Japanese media is emotionality (see [1]). Emotionality in Japan is related to the “culture of sympathy” [14], a bonding tool that is used to achieve interdependence between people. Sharing emotional states with others is perceived as important to understanding others [1]. Markus et al. [1] analyzed Olympic media coverage and found that, compared with American media coverage, the Japanese media depicted more emotional “tearjerkers” detailing the hardships that athletes had overcome. Thus, we expected that during the disaster period, attention might have been given not only to objectivity, but also to the emotionality of the shared experience of sadness or anger.

### Present Study

Our purpose in this study was to ascertain the level of objectivity and emotionality in the media coverage of this national disaster in Japan. Objectivity-seeking might be one important factor in journalism, but in the socio-cultural context of Japan, emotionality might also be present. If so, which element were journalists emphasizing: objectivity or emotionality? And how were these two types of orientations (objectivity and emotionality) related to each other?

In Study 1, we conducted an exploratory analysis of the content of media coverage from one TV news program (NHK) and two Japanese newspapers with nationwide circulation that were published during the six month period immediately following the disaster (from March 11^th^ to September 30^th^, 2011). Study 1 was an exploratory study, but we had two viewpoints for analysis. First, we examined whether the Japanese media would attempt to seek a balanced emotional valence (seeking neutrality) to attain objectivity, even in such negative and chaotic circumstances; thus, we investigated how often neutral facts were reported (Analysis 1: A1). In addition, we examined how the media coverage portrayed objective and emotional information and whether “factual reports” or “commentaries about the facts” contained either objective or emotional information (Analysis 2: A2).

In Study 2, we conducted a survey of journalists asking about their strategies for seeking objectivity and how they evaluate objectivity and emotionality; our focus was on their psychological processes, particularly the journalists’ orientations toward the objectivity and emotionality of their reporting. We predicted that journalists working in a Japanese socio-cultural context seek to report both objectivity and emotionality (H1), but evaluate the objectivity of articles written in chaotic conditions as not meeting their expected standards (H2). We also focused on the negative emotions they experienced during their journalistic investigations and the effects that they subsequently had on how they evaluated the objectivity of their reporting (H3).

In these two studies, we tried to identify how the news broadcasts and articles contained objective factual information and emotional reactions, and how the psychological processes of journalists affected the creation of the reports and their evaluations of the news broadcasts and articles.

## Study 1

### Method

We collected, coded and analyzed nationwide coverage of the Great East Japan Earthquake from one television channel and two newspapers dating from the period of March to September 2011. We chose the program “News Watch 9” (aired weekdays, from 9 to 10pm) on the channel “NHK,” which had a large audience share (16.8% during April 2011), the second highest for the month after the earthquake. The highest-ranking program was “News 7” (17.9%) on NHK (aired weekdays, from 7 to 7:30pm), but because this program was shorter and had the same content, we selected “News Watch 9.” We also collected print coverage from the “*Asahi*” and “*Yomiuri*” newspapers since they ranked first and second for nationwide circulation. We only analyzed “news” articles, excluding longer arguments and interpretative reports in order to establish equivalence with the TV program. During the initial period after March 11^th^, the news was not reported on the regular News Watch 9 program, but was reported around the clock in continual coverage; we therefore analyzed this coverage (broadcast by NHK) at the same time as News Watch 9 (from 9 to 10pm).

The first segment of coverage that we looked at was from March 12 to 31, the month following the earthquake; the second was from the month of April, the second month after the earthquake; the third segment was from May to June; and the fourth was from August to September. Within each segment, we only analyzed news that was broadcast or published on Mondays, Wednesdays and Fridays in order to avoid overlap, and also because of the time-saving for doing a large volume of content analysis.

To develop an inductive coding scheme, three Japanese adults who were unaware of the purpose of the study watched selections from television news programs (excluding News Watch 9). The coders were asked to catalogue and categorize all descriptions or analyses of the earthquake and nuclear accident as either factual reports or commentaries. A discussion of the topics of coverage generated by the coders yielded (1) seven conceptually distinct topics of coverage: the government, the electric company and nuclear power plant, other business enterprises, the tsunami-afflicted Tohoku area in general, other areas of Japan, areas outside of Japan, and people assisting in aid efforts, such as rescue or volunteer workers; (2) five categories of commentators: victims, specialists, journalists, aid workers/rescue personnel and government people and (3) three valences: positive, negative and neutral. Coding categories are shown in Table 1 and Table 2. Ten Japanese coders independently coded each sentence of the video segments and newspaper articles (following [1]), judging whether each sentence mentioned one or more topics included in the coding scheme and noting the topics of coverage mentioned in each case. For example, if the TV reporters said, “The tsunami victims are suffering so much. The electric company has not yet solved the nuclear power plant problem,” we coded a score of 1 for both “tsunami victims” and “the electric company and nuclear power plant.” During the practice session (for which we used ten episodes of News Watch 9 which were not included in the analysis), coders were trained to share their understandings of the coding scheme. The coding results showed a good rate of consistency (78%–91%). Twenty percent of the coverage was coded by at least two people.

**Table 1:** Coding categories and prevalence in TV program contents (percentage).

**Factual report**				
	**Valence**			
**Topics**	**Positive**	**Neutral**	**Negative**	**Total**
**Government**	0.0	2.6	0.8	3.4
**Electronic company/Nuclear power plant**	0.0	5.8	6.1	11.9
**Other business enterprises**	0.1	0.7	0.3	1.1
**Tsunami-afflicted Tohoku area**	0.4	11.0	12.4	23.8
**Other areas in Japan**	0.1	1.9	0.8	2.8
**Outside of Japan**	0.2	1.3	0.4	1.9
**Aid workers/rescue personnel**	0.1	2.8	0.8	3.7
**Other categories**	0.0	1.5	1.0	2.5
**Total**	0.9	27.6	22.6	51.1
**Commentary on reported fact**			
	**Valence**			
**Topics**	**Positive**	**Neutral**	**Negative**	**Total**
**Victims**	1.8	3.7	7.7	13.2
**Specialists**	0.3	6.3	3.2	9.8
**Journalists**	1.0	5.9	7.4	14.3
**Aid workers/rescue personnel**	0.4	0.9	1.1	2.4
**Government-related people**	0.0	0.1	0.1	0.2
**Others**	0.2	0.3	0.4	0.9
**Total**	3.7	17.2	19.9	40.8

**Table 2:** Coding categories and prevalence in newspaper contents (percentage).

**Factual report**				
	**Valence**			
**Topics**	**Positive**	**Neutral**	**Negative**	**Total**
**Government**	0.1	8.2	2.1	10.4
**Electronic company/Nuclear power plant**	0.0	6.2	5.1	11.3
**Other business enterprises**	0.1	4.0	2.2	6.3
**Tsunami-afflicted Tohoku area**	0.6	15.0	14.6	30.2
**Other areas in Japan**	0.0	1.9	1.2	3.1
**Outside of Japan**	0.4	2.5	0.8	3.7
**Aid worker**	0.3	4.0	0.6	4.9
**Other categories**	0.1	0.8	0.3	1.2
**Total**	1.6	42.6	26.9	71.1
**Commentary on reported facts**				
	**Valence**			
**Topics**	**Positive**	**Neutral**	**Negative**	**Total**
**Victims**	0.9	4.7	4.5	10.1
**Specialists**	0.1	2.0	1.2	3.3
**Journalists**	0.0	1.4	1.4	2.9
**Aid workers/rescue personnel**	0.2	0.8	0.3	1.3
**Government-related people**	0.0	0.8	0.5	0.2
**Others**	0.2	0.4	0.2	1.8
**Total**	1.4	10.1	8.1	19.6

### Coding Categories

First, each sentence was categorized as either a “factual report” or a “commentary on reported facts.” If a sentence was categorized as the former, the coder selected the topic of the factual report. The valence of the reported fact (positive, neutral or negative) was also coded. If a sentence was categorized as a comment on reported facts, we coded the commentator (victim, specialist, journalist, aid worker / rescue personnel, or government person) and valence (positive, negative or neutral).

### Results

Tables 1 and 2 show how frequently each topic was coded in the media coverage.

#### Factual Reports

We analyzed the two types of media (TV and newspaper) separately (Table 1 shows the results from TV coverage and Table 2 shows the results from newspapers). Factual reports made up 51.1% of the news on TV programs; of this, 22.6% was made up of negative factual information and 27.6% was made up of neutral factual information, a considerable amount even under such adverse circumstances. Regarding topics, coverage on the tsunami-afflicted Tohoku area and the electric company contained both negative factual reports (12.4% and 6.1% of all content, respectively) and neutral factual reports (11.0% and 5.8% of all content, respectively). Reports on the government had more neutral (2.6% of all content) than negative news (0.8%).

Factual reports made up 71.1% of newspaper articles, a higher rate than in the TV coverage. Furthermore, a large percentage of the content was made up of neutral factual reports (42.6% of all descriptions) and a smaller percentage was negative (26.9%). Neutral information was more likely to be reported across all topics of coverage.

#### Commentaries

In the TV programs, commentaries made up 40.8% of all content. A majority of the commentaries were delivered by journalists (14.3%), followed by victims (13.2%) and specialists (e.g., nuclear scientists, 9.8%). In the TV programs, 19.9% of the commentaries were negative and 17.2% were neutral. Thus, the rate of negativity was higher than the rate of neutrality for commentaries as opposed to factual reports. Journalists (e.g., newscasters), victims and aid workers/rescue personnel gave more negative than neutral commentaries. On the other hand, specialists and government officials gave more neutral than negative commentaries.

With regard to newspapers, 19.6% of all content was comprised of commentaries. One reason for such a low percentage may have been that commentaries in newspapers tended to appear in argumentative essays and longer articles that were excluded from the study because they were not news reports, while the TV coverage included brief commentaries alongside factual reports. Most commentaries in newspapers were by victims (10.1% of all content), followed by specialists (3.3%) and journalists (2.9%). In newspapers, 8.1% of commentaries were negative and 10.1% were neutral. Thus, there were fewer negative commentaries in newspapers than in the TV programs.

### Discussion

The analysis revealed that the media coverage of the Great East Japan Earthquake sought to maintain objectivity by conveying neutral information in considerably chaotic circumstances, especially in the newspaper articles (A1). Inconsistent information, such as presenting neutral factual reports alongside negative commentaries, was also conveyed (A2). However, such negative commentaries appeared more often in TV programs than in newspaper articles. This might be because we excluded “commentary” articles—which would likely include emotional comments—from our analysis of newspapers; thus only TV programs showed informational inconsistency in containing both factual reports and commentaries. However, it could be argued that the TV programs contained both factual reports and commentaries within the same story, rather than separately, as in the newspaper articles. In fact, such a co-occurrence of neutral and emotional information in the same report could have affected audiences’ perceptions of the reports [15].

Even within the adverse conditions surrounding the events of March 11^th^, media coverage included neutral information for both the nuclear accident and the tsunami-afflicted areas. As such, it is important to investigate whether this was due to the journalists’ attempts to maintain objectivity. Study 2 was designed to clarify this point. In Study 2 we selected two topics (the nuclear accident and the tsunami-afflicted Tohoku area) about which reports were shown to be more factually-oriented than those regarding other topics.

## Study 2

In Study 1, we found that the media coverage of the Great East Japan Earthquake was mostly neutral. Since the news was produced by journalists, we attempted to examine the extent to which journalists explicitly expressed an orientation towards either objectivity or emotionality, as well as the intentionality behind their news coverage, e.g., emphasizing or omitting content. Additionally, we were interested in their evaluations of the objectivity and emotionality of competing coverage. In the content analysis, the coverage of the nuclear power plant accident and the tsunami-afflicted Tohoku area had similar patterns of valence. Therefore, we predicted that journalists generally seek to be objective as part of their work, as opposed to seeking emotionality (H1), as shown in Study 1’s finding that the objectivity of the media coverage was similar for both the nuclear accident and tsunami-afflicted areas. However, the characteristics of coverage on the nuclear accident and the tsunami-afflicted areas were different. Whereas people might require objective information about the former to make critical decisions [4], in the latter case emotional information about the tsunami-afflicted Tohoku area might be conveyed in order to elicit an emotional response in people living in non-afflicted areas (e.g., increasing sympathy to incite aid) in the Japanese socio-cultural context (see [7]). Thus we predicted that there would be an interaction effect wherein orientations towards objectivity would be greater for coverage of the nuclear power plant than for coverage of the tsunami-afflicted Tohoku area.

In addition, we predicted that, particularly with regard to the nuclear accident, journalists’ evaluations of objectivity would judge it as falling short of their expected standards (H2), since the objective information that the public required would be complicated by the nature of the accident, which was the first of its kind in both complexity and scale.

Furthermore, journalists might experience negative emotions (including trauma) during their investigative work (see [16]), and such emotional reactions might be related to their evaluations of the objectivity of their articles (H3).

### Method

The survey study was approved by the Kokoro Research Center at Kyoto University following the Japanese Psychological Association guidelines. We made an online web survey (using “surveymonkey”) so that anonymous participants could easily access and complete the questionnaire. The URL of the survey website was announced and participants were provided written informed consent agreements to participate in this study. They provided consent by completing the survey (the consent button was a required field on the web survey). This procedure was approved by each news organization (e.g., newspaper company) that agreed to recruit potential participants.

#### Participants

First, the authors sent the link of the online survey to journalists we personally knew and asked them to complete the online questionnaire and/or to send the survey link to their colleagues. We also asked five mass media organizations (including TV broadcasting companies and newspaper-related organizations) to participate in this study, and three of them agreed to recruit participants by sending details of the survey to journalists belonging to their organization. In total, we asked 44 individuals and three organizations to respond to or distribute the web-based survey link to journalists. The participants of this study were 115 Japanese journalists (55 male, 13 female, 47 uncategorized), who ranged in age from 23 to 70 years (the majority were in their 30s) and worked at broadcasting companies, newspaper publishing companies, or news service agencies. They completed the survey between March and April 2012 (a year after the earthquake). Of the 115 participants, we used 98 who reported on either the nuclear power plant, the tsunami-afflicted Tohoku area, or both for the main analysis. A total of 62 participants (53.9%) covered both the nuclear power plant accident and the tsunami-afflicted Tohoku area, 17 participants (14.8%) reported only on the nuclear accident, and 19 (16.5%) on only the tsunami-afflicted Tohoku area.

We asked them to report what type of organization they worked for (TV or newspaper) but 42.6% did not answer this question. Thus we were unable to do a comparison of responses based on the media type.

#### Survey

The questionnaire consisted of four parts: (1) the journalists’ investigative activities and their evaluation of their coverage of the nuclear power plant accident, (2) their activities and their evaluation of their coverage of the tsunami-afflicted Tohoku area, (3) their emotions during their investigative work and (4) their attitudes toward journalism in general and a free description of the earthquake coverage and the media in general. We first asked whether they were engaged in covering (writing/reporting on) the nuclear accident and the tsunami-afflicted Tohoku area, and those who answered “yes” completed part 1, part 2, or both (if applicable), and part 3. All participants completed part 4.

#### Orientation

For items in parts 1 and 2—which were scored on a 5-point Likert scale ranging from 1 (not at all) to 5 (very much)—we asked about their orientations toward objectivity (2 items) and audience emotion (4 items) for each topic of coverage (the nuclear power plant accident and the tsunami-afflicted Tohoku area). Items for orientation toward objectivity were “try to maintain a balance when introducing commentaries” and “avoid having only positive or negative information in coverage.” Since the correlations between these two items were sufficiently high (.52 and. 62 for nuclear power plant and the tsunami-afflicted Tohoku area coverage, respectively), we calculated the average scores of the two items. Orientation towards audience emotion (orientation toward emotionality) questions were “try to elicit joy or hope”, “try to show the tragedy of the event”, “try to elicit crisis awareness” and “try to reduce anxiety.” Cronbach’s alpha coefficients were. 61 and. 64 for the nuclear accident and the tsunami-afflicted Tohoku area, respectively, and we calculated the mean scores.

#### Evaluation

We asked participants the extent to which their coverage reflected (1) objective facts, (2) personal opinions, (3) the opinions of the organization they belonged to, (4) specialists’ opinions and (5) victims’ opinions, using a 4-point scale (1 = not at all, 2 = somewhat, 3 = considerably, 4 = almost entirely), for both types of coverage (the nuclear power plant accident and the tsunami-afflicted Tohoku area). We calculated the average scores for both types of coverage since they were highly correlated, and the result was no different than when they were examined separately in the pre-analysis.

A second 4-point objectivity evaluation scale (1 = not at all, 2 = not very much, 3 = somewhat, 4 = very much) measured the objectivity of the coverage (3 items). The items were “to what extent did the broadcast coverage you were involved in adhere to the facts”, “to what extent was the coverage more objective than emotional” and “to what extent did the coverage reflect the real situations you experienced during your investigative work?” Since this scale had only three items, the Cronbach’s alpha coefficient for each topic of coverage was relatively low (.55 and. 50 for the nuclear power plant and the tsunami-afflicted Tohoku area coverage, respectively) but we calculated the mean scores for each topic of coverage since preliminary analysis showed that the pattern of the results was similar when we did same analysis with each item.

We also asked participants whether their organizations preferred to cover certain topics, for which possible answers were “yes”, “it is likely”, “no” and “I don’t know.”

#### Emotion

Participants rated the intensity of the emotions they experienced from 1 (did not feel at all) to 5 (felt very intensely) for both negative and positive emotions. A factor analysis revealed three factors: positive emotions (pride, hope, joy, respect, friendliness; α =. 77), negative emotions (resentment, anger, pity, desperation, hopelessness, anxiety, sadness; α =. 87) and guilt-related emotions specific to this situation (indebtedness, guilt, feelings of restraint toward the interviewee, loneliness, pity; α =. 86). We calculated the mean scores for each domain of emotion.

#### Perception of the Japanese Media as Having an Emotional Presentation Style

We asked participants about their general attitudes toward the “emotionality” of media coverage in Japan (6 items, 1 = completely disagree, 5 = completely agree). The items were “in general, I think the majority of news coverage in Japan caters to the audience’s preferences”, “generally speaking, audiences prefer stories that are sad or are about overcoming hardship over happy stories”, “in general, news that relates the emotional aspects of a situation is important in coverage”, “someone’s story of hardship is preferred over someone’s story of honor as a topic of news coverage”, “for disaster coverage, the media tends to convey more negative emotional material such as misery or hardship” and “most disaster coverage is constructed using a story where someone has overcome some form of hardship.” The Cronbach’s alpha coefficient of these items was. 76. We calculated the mean scores.

#### Free Description

At the end of the survey, we asked participants to freely describe their emotions or thoughts about their investigative activities and the emotions they experienced during their work reporting on the earthquake.

### Results

#### Post-earthquake Investigative Coverage Activities

Of those covering the nuclear accident, the majority of their investigative work was conducted in Fukushima, followed by Tokyo. The 98 journalists who reported on either the nuclear power plant, the tsunami-afflicted Tohoku area, or both were the most likely to interact with disaster victims or local government officials in the affected areas, followed by risk managers from the government, Tokyo Electric Power Company (TEPCO) and the Fukushima Daiichi nuclear power plant. For the tsunami-afflicted Tohoku area, investigative work was primarily conducted in Miyagi, which was severely damaged by the tsunami (26.1% went there to cover the nuclear accident in Fukushima and 49.5% went there to cover the tsunami-afflicted area), followed by Fukushima (48.9% went there to cover the nuclear accident and 22.2% went there to cover the tsunami-afflicted area) and Iwate (19.6% went there to cover the nuclear accident and 39.5% went there to cover the tsunami-afflicted area). The greatest number of interviewees for the nuclear accident were local government officials in the afflicted areas (45.7%) and disaster victims (41.3%), followed by officials from the Japanese government (30.4%) and TEPCO, which owned the Fukushima Daiichi nuclear power plant (27.2%). The greatest number of interviewees in the tsunami afflicted areas were disaster victims (61.7%) and local government officials in the afflicted areas (54.3%), followed by aid workers (46.9%). The most frequently stated reason (37.1%) for why they conducted interviews in the tsunami-afflicted Tohoku area was “both because of their own personal desire and also at the request of the organization they work for.”

#### Orientation and Comparison of the Coverage of the Nuclear Power Plant and the Tsunami-afflicted Tohoku Area

**Fig 1:**
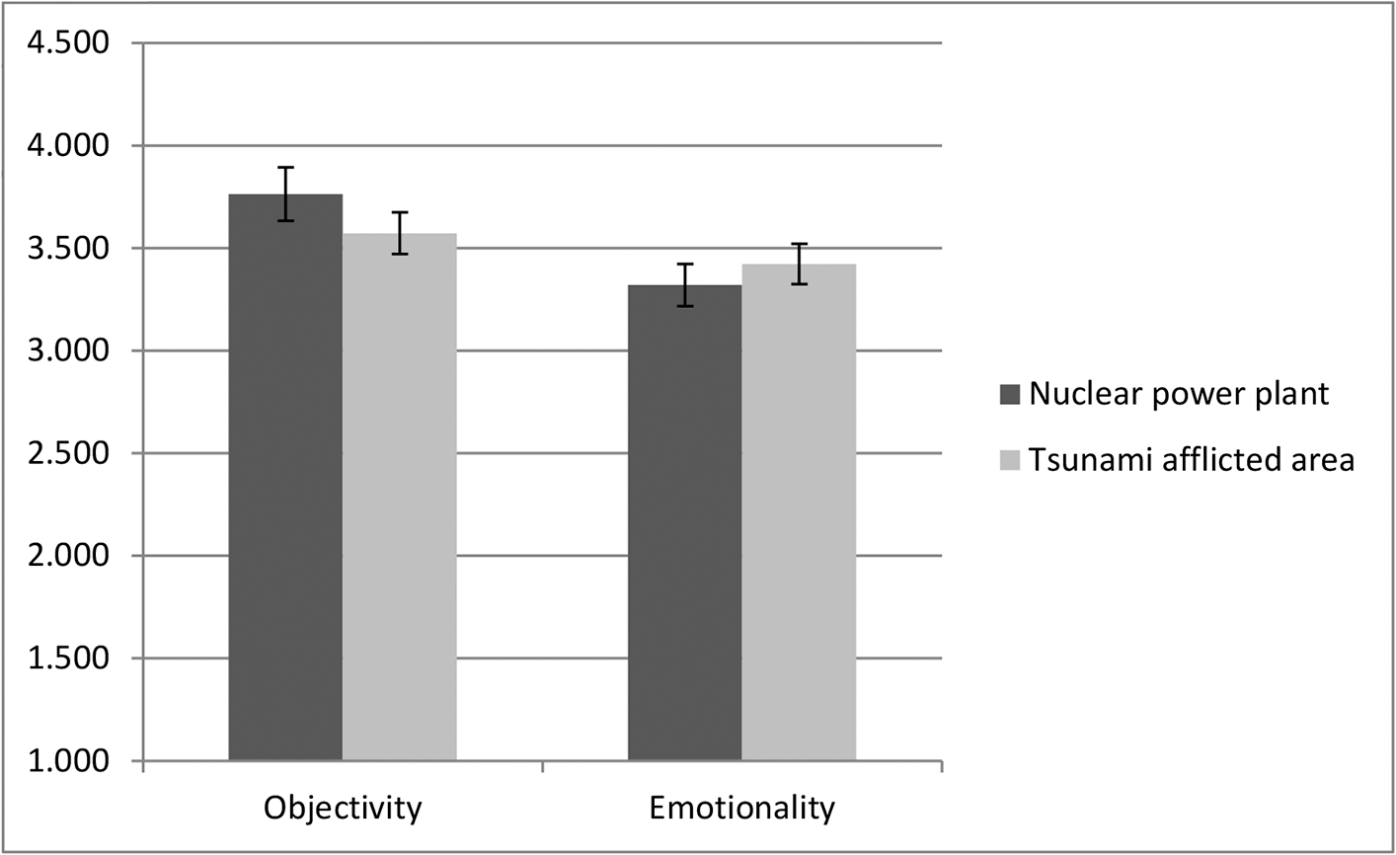
Journalists’ orientations toward objectivity and emotionality in the coverage of the nuclear power plant accident and the tsunami-afflicted Tohoku area.

Overall, the orientation toward objectivity was above the mid-point for both topics of coverage (*t*s > 5.6, *p*s <. 001, *r*s >.57). The mean scores of orientation toward objectivity in the coverage of the nuclear power plant and the tsunami-afflicted Tohoku area were 3.77 (*SD* = 0.98) and 3.53 (*SD* = 0.77), respectively. The correlation between these scores was significant (*r* =. 35, *p* <. 01); therefore, journalists who were objectively oriented tried to be somewhat consistent in their attitudes towards both topics of coverage. An orientation towards emotionality was above the mid-point for both topics of coverage (*t*s > 3.91, *p*s <. 001, *r*s >. 41). The mean score for emotionality orientation for the nuclear power plant coverage was 3.34 (*SD* = 0.76), and was 3.41 (*SD* = 0.77) for the tsunami-afflicted Tohoku area coverage. The correlation between these scores was significant (*r* =. 63, *p* <. 001), implying that those who displayed an orientation toward emotionality tried to be consistent in their attitudes for both topics of coverage. A 2 (topic of coverage: nuclear power plant vs. the tsunami-afflicted Tohoku area) × 2 (orientation: objectivity vs. emotionality) x 3 (gender: male, female, unknown) mixed ANOVA for journalists who answered orientation questions for both topics of coverage revealed a main effect of orientation (*F*(1, 52) = 8.00, *p* <. 01, partial *η* ^2^ =. 13); specifically, journalists were more likely to be oriented towards objectivity than towards emotionality. A significant interaction effect was found, indicating that the difference between an objective and an emotional orientation was larger for the coverage of the nuclear power plant than for the coverage of the tsunami-afflicted Tohoku area (*F*(1,52) = 4.64, *p* <. 04, partial *η* ^2^ =. 08; Fig 1). There was no gender difference.

Furthermore, an orientation toward objectivity and emotionality were both positively correlated with coverage of the tsunami-afflicted Tohoku areas (*r* =. 29, *p* <.02), but this was not the case for coverage of the nuclear plant accident (*r* = -.19, *p <*.*11*). This result suggests that for topics for which journalists were more likely to express emotionality, their perceptions of objectivity and emotionality were not separated. However, for topics for which they sought objectivity more strongly, they felt objectivity to be independent of emotionality.

#### Evaluation

Participants evaluated the coverage as predominantly reflecting objective facts (*M* = 3.29, *SD* = 0.58), followed by victims’ opinions (*M* = 2.84, *SD* = 0.66), specialists’ opinions (*M* = 2.39, *SD* = 0.60), personal opinions (*M* = 2.20, *SD* = 060) and the opinions of the organizations to which they belonged (*M* = 1.39, *SD* = 0.59). All differences between categories were significant (*F* (4, 31) = 27.52, p<.001, partial *η* ^2^ =. 78), except for the differences between journalists’ personal opinions and the opinions of their organizations. Thus, their evaluations of the broadcast or published coverage suggest that, from their perspective, their coverage was more likely to reflect reality and less likely to be influenced by “pressure” from their news organizations.

As predicted, evaluations about objectivity were higher for the coverage of the tsunami-afflicted Tohoku area (*M* = 3.08, *SD* = 0.55) than for coverage of the nuclear accident (*M* = 2.96, *SD* = 0.63). A within-subjects t-test for participants who reported on both topics of coverage was significant (*t*(53) = 2.19, *p* <. 04, *r* =. 29): that is, journalists were more confident about the objectivity of the coverage of the tsunami-afflicted Tohoku area than they were about the coverage of the nuclear accident. Specifically, 35% of the participants evaluated the published and broadcast coverage of the nuclear accident as being incapable of adhering to the facts. This implies that they felt they could not fully comprehend the scope of the accident because the facts were too complicated to obtain all the information necessary for completely objective coverage.

A correlation analysis was conducted to investigate the extent to which participants’ orientations toward objectivity and emotionality were related to their evaluations of news coverage and their perceptions of the Japanese media. Results revealed that both orientations (objectivity and emotionality) for coverage of the tsunami-afflicted Tohoku area were positively correlated with responses stating that the coverage reflected facts (*r* =. 27 and. 29, *p*s <. 03) and participants’ evaluations of the objectivity of the coverage of the tsunami-afflicted Tohoku area (*r* =. 41, *p* <. 001 and *r* =. 29, *p* <. 02). Thus, journalists believed that depicting the reality of the situation accurately was related to both an orientation toward objectivity and emotionality when covering the tsunami-afflicted Tohoku area. However, the results for the coverage of the nuclear accident were mixed. We found that having an orientation toward emotionality was positively correlated with the inclusion of victims’ opinions in coverage (*r* =. 40, *p* <. 03) and a perception of the Japanese media as using emotionality in their stories (*r* =. 24, *p* <. 07) when covering the nuclear accident; this suggests that emotionality was associated with the journalists’ perspectives on the Japanese media’s emotionality and was affected by victim opinions. Furthermore, evaluations of objectivity for the nuclear accident coverage were not significantly correlated with accurately reflecting reality or with having an orientation toward objectivity when covering the accident (*r*s <. 08, ns). Thus, in coverage of the nuclear accident, journalists’ attitudes toward objectivity were not related to their evaluations of the objectivity of the news coverage, suggesting that they experienced ambivalence and waning confidence in the nuclear coverage.

With regard to whether journalists believed their organizations preferred particular topics, 29.6% answered “yes” and 10.4% answered “it is likely:” thus, in total, 40% believed that there was a preference for topics. Only 3.5% answered “no,” 15.7% answered “I don’t know,” and 40.9% did not answer the question. These results suggest that a preference for particular content existed to some extent, and that journalists were aware of this preference.

#### Emotion

Journalists were more likely to feel negative emotions (*M* = 3.73, *SD* = 0.92), followed by guilt-related emotions (*M* = 2.91, *SD* = 1.15), and less likely to feel positive emotions (*M* = 2.61, *SD* = 0.89). The differences between each valence were significant (*F*(2,142) = 31.50, *p* <. 0001, partial *η* ^2^ =. 31); a post hoc test indicated that negative emotions were felt more intensely than were other types. Among negative emotions, sadness was felt most intensely (*M* = 4.4). This pattern did not differ between those who were involved in the coverage of the nuclear power plant and those who were not, or between those who were involved in the coverage of the tsunami-afflicted Tohoku area and those who were not. Negative and guilt-related emotions were positively correlated with journalists’ evaluations of their own coverage as reflecting their personal opinion (*r* =. 30, *p* <. 02 and *r* =. 20, *p* <. 09, respectively); furthermore, both types of emotion were negatively correlated with their evaluations of their own coverage as reflecting the facts (*r* = -.20, *p* <. 11 and *r* = -.36, *p* <. 002), evaluations of objectivity for coverage of the nuclear accident (*r* = -35 and-.38, *p*s <. 005) and evaluations of objectivity for coverage of the tsunami-afflicted Tohoku area (*r* = -35 and-.46, *p*s <. 004, respectively). We could not identify a causal relationship; however, the journalists’ experiences of negative or guilt-related emotions during their fieldwork may have influenced their opinions and coverage, leading to an overall reduction of objectivity in their reporting.

#### Regression Analysis for the Evaluation of Objectivity

To examine what was predictive of an evaluation of objectivity for the coverage, we performed a regression analysis on the analysis of objectivity for the coverage of both the nuclear power plant accident and the tsunami-afflicted Tohoku area. The dependent variables were guilt-related emotion (since it had a stronger correlation coefficient to objectivity evaluation than the other two emotions), orientation toward objectivity and emotionality (see Table 3). For the coverage of the nuclear accident, the evaluation of objectivity was negatively related with guilt-related emotions (*β* = -.37, *p* <. 004). For the coverage of the tsunami-afflicted Tohoku area, an evaluation of objectivity was negatively related to guilt-related emotions (*β* = -.38, *p* <. 001) and positively related to an orientation toward objectivity (*β* =. 25, *p* <. 03). Again, we could not identify a causal relationship; however, journalists’ guilt may have reduced the objectivity of their coverage or, conversely, lowered objectivity may have resulted in increased guilt.

**Table 3:** Regression analysis for the evaluation of objectivity of the broadcast and published news reports.

		Target article: Nuclear power plant accident			Target article: Tsunami-afflicted area		
		*β*	*t*	*p*	*β*	*t*	*p*
**Guilt- related emotion**		-.37	-3.01	.004	-.38	-3.46	.001
**Objectivity orientation**		.10	0.79	n.s.	.25	2.22	.03
**Emotionality orientation**		.10	0.81	n.s.	.18	1.60	.114.
***R*** ^**2**^		.19			.32		

#### Free Description

Forty-one participants (36% of the 115 participants) provided free-format descriptions. Participants expressed resentment about the nuclear power plant accident (9.8% of those who provided a free description), difficulty to understand the facts of the situation (7.32%), and a feeling of responsibility for the decreasing trustworthiness of the media, such as the spreading of harmful rumors about Fukushima (14.6%). As for the tsunami-afflicted Tohoku area, they expressed having low confidence in the accuracy of coverage (22.0%), feelings of helplessness in trying to help the victims (12.2%), a sense of responsibility to continue reporting on the tsunami-afflicted area (17.1%) and ambivalence toward the objectivity of their coverage (14.6%). In addition, 7.31% of those who wrote a free description reported experiencing PTSD (see [15]) and described their own experience as victims.

### Discussion

Study 2 suggests that the journalists’ were more likely to be objectively-oriented rather than emotionally-orientated to the nuclear power plant accident and the tsunami-afflicted Tohoku area (H1).

While journalists’ attitudes toward objectivity were not related to their evaluation of the objectivity of the coverage of the nuclear accident, they were related for the coverage of tsunami-afflicted area. An objectivity orientation might have increased their evaluation of articles about the tsunami-afflicted area, but this was not the case for coverage of the nuclear accident, suggesting that the accident was too difficult to be objective about, even if the journalist tried to be.

Interestingly, for the coverage of the tsunami-afflicted area, orientations toward objectivity and emotionality were positively correlated. Thus, for Japanese journalists, the concept of emotionality does not conflict with objectivity when they feel they have allowances to write emotionally evocative articles. This might reflect a Japanese socio-cultural context in journalism that requires both objective facts and emotional comments to evoke sympathy [1]. However, this pattern was not found in the nuclear power plant coverage, where journalists felt a strong orientation toward objectivity. For this type of coverage, orientations towards objectivity and emotionality were fairly negatively correlated (though this effect was not significant).

Participants’ evaluations of objectivity were higher for the coverage of the tsunami-afflicted Tohoku area than for coverage of the nuclear accident. This suggests that the journalists lacked confidence in dealing with such a novel and large-scale accident (H2). They also noticed that the content that was preferred by the media organizations changed with time. They experienced strong feelings of guilt as a result of their investigative work, which may have resulted in decreased objectivity in their reporting (H3). In particular, guilt-related emotions reduced their evaluation of the objectivity of the articles, both for the nuclear power plant accident and tsunami-afflicted area.

Thus, in line with our hypotheses, journalists sought objectivity, but in circumstances such as those present in the aftermath of the Great East Japan Earthquake, they evaluated their objectivity as not sufficiently meeting their expectations, especially for coverage of the nuclear power plant accident,.

## General Discussion

As we presumed that the media plays an important role in constructing socio-culturally shared realities, we analyzed media coverage during the aftermath of the massive earthquake in Japan and investigated how such realities were shown to the audience and constructed by journalists. Thus, we need to consider the psychological aspects of the coverage, as such coverage is not just a macro-level phenomenon, but is constructed through micro-level phenomena by journalists under the influence of psychological functions (e.g., motivation, emotion and cognition).

Through an analysis of media coverage and a survey of journalists, we found that the content of the coverage consisted of neutral factual reports, even in the rather chaotic situation that existed during the first six months after the March 11^th^ triple earthquake-tsunami-nuclear disaster. We examined how the journalists evaluated the objectivity and emotionality of their work in Study 2 and found that journalists sought to maintain objectivity, as shown in the broadcast reports and published articles that we examined in Study 1.

Though the broadcast coverage contained neutral information, the journalists’ attitudes toward the reporting were somewhat ambivalent, especially with regard to the nuclear accident. For the coverage of tsunami-afflicted area, they simultaneously sought both objectivity and emotionality, which might be the default attitude in Japanese socio-cultural constructions. In contrast, for the coverage of the nuclear power plant accident, they felt a stronger orientation towards objectivity than emotionality, and those two orientations showed a fairly negative correlation. Furthermore, the journalists felt less confident about the objectivity of their reporting on the nuclear power plant accident, especially where they felt guilt-related emotions. Study 1 suggested that although the broadcast reports and published articles were fairly neutral, the journalists were not fully satisfied with their objectivity.

Actually, Niwa and Fujita [9] have suggested that from a journalist’s perspective, the Great East Japan Earthquake was distinct in the extent, complexity and enormity of the disaster, which was beyond comparison due to the simultaneity of the earthquake, tsunami and nuclear accident. Therefore, journalists were unsure of how to report on this multi-faceted event ([4–5]). In particular, this kind of nuclear accident was a first for Japan and it was difficult for even science journalists to obtain concrete objective information. As a result of this journalistic bewilderment, lay people felt strong distrust toward the media, politicians, and even nuclear power experts ([17]). Some might have suspected these experts of having connections with the nuclear plant company (TEPCO) that were leading them to express positive attitudes to protect TEPCO’s image. Future research should investigate whether these phenomena are observable only in Japan, or if they are widely shared across cultures after disasters. As previous research has suggested ([13] [1]), a comparison of the media coverage of the Great East Japan Earthquake by agencies in Japan and other countries (e.g., *The New York Times* and BBC News) may contribute to an understanding of the cultural differences and similarities in the coverage of a specific tragic event. Indeed, a previous study has suggested that the disaster was treated as major news both in the US and Japan (but not in some other countries), although the content and communication style differed [18].

Our findings are limited by the fact that our analysis only looked at media coverage from a six-month period and examined news from only one TV program. An analysis covering a longer span of time and involving several programs would improve generalizability. In a follow-up study, how the media conveyed information related to the Great East Japan Earthquake after an extended period could also be examined. In Study 2, we were unable to compare responses from the TV and newspaper journalists because many participants did not answer the questions about their respective organizations; thus, future studies that made such comparisons could yield more robust results.

Media analyses from a sociological perspective sometimes treat the media as “collective” phenomena; however, content is created by individual journalists and reflects their orientation and emotional states. As such, media coverage is not just a collective or macro phenomenon, but is also comprised of individual psychological factors.
